# Friends or Foes? Rapid Determination of Dissimilar Colistin and Ciprofloxacin Antagonism of *Pseudomonas aeruginosa* Phages

**DOI:** 10.3390/ph14111162

**Published:** 2021-11-15

**Authors:** Katarzyna M. Danis-Wlodarczyk, Alice Cai, Anna Chen, Marissa R. Gittrich, Matthew B. Sullivan, Daniel J. Wozniak, Stephen T. Abedon

**Affiliations:** 1 Department of Microbial Infection and Immunity, The Ohio State University, Columbus, OH 43210, USA; danis-wlodarczyk.1@osu.edu; 2 Department of Microbiology, The Ohio State University, Columbus, OH 43210, USA; cai.712@buckeyemail.osu.edu (A.C.); chen.7295@osu.edu (A.C.); gittrich.1@buckeyemail.osu.edu (M.R.G.); sullivan.948@osu.edu (M.B.S.); 3 Department of Civil, Environmental and Geodetic Engineering, The Ohio State University, Columbus, OH 43210, USA

**Keywords:** antibacterial therapy, bacteriophage therapy, ciprofloxacin, colistin, phage PEV2, phage ΦKMV, phage LUZ19

## Abstract

Phage therapy is a century-old technique employing viruses (phages) to treat bacterial infections, and in the clinic it is often used in combination with antibiotics. Antibiotics, however, interfere with critical bacterial metabolic activities that can be required by phages. Explicit testing of antibiotic antagonism of phage infection activities, though, is not a common feature of phage therapy studies. Here we use optical density-based ‘lysis-profile’ assays to assess the impact of two antibiotics, colistin and ciprofloxacin, on the bactericidal, bacteriolytic, and new-virion-production activities of three *Pseudomonas aeruginosa* phages. Though phages and antibiotics in combination are more potent in killing *P. aeruginosa* than either acting alone, colistin nevertheless substantially interferes with phage bacteriolytic and virion-production activities even at its minimum inhibitory concentration (1× MIC). Ciprofloxacin, by contrast, has little anti-phage impact at 1× or 3× MIC. We corroborate these results with more traditional measures, particularly colony-forming units, plaque-forming units, and one-step growth experiments. Our results suggest that ciprofloxacin could be useful as a concurrent phage therapy co-treatment especially when phage replication is required for treatment success. Lysis-profile assays also appear to be useful, fast, and high-throughput means of assessing antibiotic antagonism of phage infection activities.

## 1. Introduction

“*It’s not about phages or antibiotics. It’s about phages and antibiotics.*” Paul Grint [1].

Development of the first antibacterial chemotherapeutics, Salvarsan [2,3], Prontosil [4], and penicillin [5], transformed clinical treatments of bacterial infections. Though antibiotic therapy came to represent the standard of care for the treatment of these infections, its utility is threatened by the emergence of multi/pan-drug resistant or tolerant pathogens [6,7]. This has led to increased health care expenses and economic damages that are comparable to those of the 2008–2009 global financial crisis [8].

The U.S. Centers for Disease Control and Prevention (CDC) estimates that more than 2.8 million antibiotic-resistant infections occur each year, leading to more than 35,000 deaths only in the United States [9]. Globally, this number increases to at least 700,000 deaths each year [8] and is predicted to lead to 10 million deaths per year by 2050 [10]. This is particularly concerning given the limited number of new antimicrobial agents, both currently available and in drug development pipelines, that are active especially against Gram-negative pathogens [11]. Moreover, infections by even antibiotic-sensitive bacteria can lead to antibiotic tolerance [12,13,14,15,16,17,18]. Thus, additional antibacterial agents and approaches are needed, with traits of interest including abilities (1) to treat antibiotic-recalcitrant bacterial infections, (2) to reduce the potential for bacteria to evolve resistance, and (3) to work in conjunction with traditional, standard-of-care antibiotics.

One emerging antibacterial approach is phage therapy [19,20]. Bacteriophages (phages) can lyse and kill bacteria and collectively are the most abundant replicative entities in the biosphere [21]. They have been in common clinical use for decades in several countries, particularly those from former the Soviet Union republics (Georgia and Russia) as well as Poland. Currently, in Europe as well as in the U.S. [22,23,24,25,26,27], new phage therapy centers are opening and several new clinical trials are taking place [28,29,30,31,32,33].

Phage therapy in the clinic is often used in concurrent combination with antibiotic treatments [26,34,35,36,37,38,39,40,41,42,43,44,45,46,47,48,49,50,51,52,53,54,55,56,57,58,59,60,61,62,63,64,65,66,67,68,69,70,71,72,73,74]. Crucial with combination therapies, however, is avoidance of substantial antagonisms between agents [75,76]. Phages, while infecting antibiotic-treated bacterial populations, ideally should retain bactericidal, bacteriolytic, and virion production activities. It can be useful, therefore, to identify possible incompatibilities between specific phages and antibiotics early during the development of combination therapies, or in the course of compassionate use [19,25,27]. There has been comparatively little analysis, though, of the antagonistic impacts of antibiotics on the ability of phages to successfully infect and lyse antibiotic-sensitive bacteria under standard, pharmacologically relevant conditions.

When testing has been completed, emphasis instead is usually on combinations of phages and sub-inhibitory antibiotic concentrations along with their impact on the growth of bacterial cultures over multiple-hour time scales or using biofilm-based instead of broth-based assessments. We feel, therefore, that it would be a useful addition to the study of phage therapy to employ pre-clinical analyses that (1) are broth based, (2) use standard media for MIC evaluation (Mueller-Hinton broth), (3) employ standard-of-care antibiotic concentrations, i.e., minimum inhibitory concentrations (MICs) or higher, (4) take place over the time scales that are roughly equivalent to those of individual phage infections, and (5) examine especially anticipated [47] antagonistic impacts of antibiotics on phage properties relative to no-antibiotic controls.

To assess the impact of antibiotics on the antibacterial activities of phages, given the substantial numbers of combinations of therapeutic phage and antibiotic types that are possible, higher-throughput approaches are needed, and ideally these would be performed under conditions that are similar to those employed to determine antibiotic MICs [77,78,79]. Here, we validate a 96-well microtiter plate-compatible turbidimetric, ‘lysis-profile’ [80] means of assessing phage infection activity in the presence and absence of antibiotics, as corroborated by other traditional but more time-consuming methods of phage infection activity determination. This was carried out using two antibiotics possessing contrasting mechanisms of action, colistin and ciprofloxacin. We find that colistin, even at low but still bacteria-inhibiting concentrations, is highly antagonistic to the infection activities of three *Pseudomonas aeruginosa* phages, PEV2 [81,82], LUZ19 [83], and φKMV [84]. In contrast, even in the presence of relatively high but still clinically relevant concentrations of ciprofloxacin, these phages retain substantial bacteriolytic and virion-production activities.

## 2. Results

**Figure 1:**
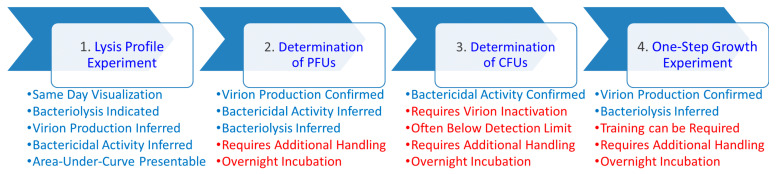
Workflow schematic with pros and cons of the different approaches indicated. Different analyses are indicated in bubbles (top), with pros (blue) and cons (red) of each approach presented beneath. These include: (**1**) Lysis profile experiments as can be performed employing a variety of phage multiplicities of infection (MOIs) and antibiotic concentrations (multiples of MICs). Immediately following individual lysis profile experiments, both (**2**) PFU (plaque-forming unit) and (**3**) CFU (colony-forming unit) determinations can be undertaken to confirm virion production activities as well as bactericidal impacts, with these latter analyses requiring both plating and overnight incubation. (**4**) Phage infection activities can then be corroborated using separate one-step growth experiments. “Requires additional handling” refers to, e.g., diluting and plating of cultures along with counting of plaques or colonies. “Training can be required” refers to the greater technical difficulty associated with performing one-step growth experiments relative to lysis-profile, PFU, or CFU determinations. Bactericidal activity is the physiological killing of bacteria, as can be assessed as reductions in colony-forming units (CFUs). In practice, such as following certain antibiotic treatments, this can occur without bacterial lysis. Bacteriolytic activity involves the physical destruction of bacterial cell envelopes as can be observed as drops in bacterial culture turbidity, but which will result in reductions in CFU counts as well. Virion production is assessed as increases in numbers of plaque-forming units (PFUs), which for lytic phages also will be associated with bacterial lysis as well as bacteria killing. See Appendix B for an explanation of how the occurrence of virion production also may be inferred from lysis profile curves.

Using a rapid, semi-automated, lysis profile-based workflow (Figure 1), we assessed the impact of two antibiotics, colistin or ciprofloxacin, on *P. aeruginosa* phage PEV2 infection activities. We were then able to confirm phage population growth and bactericidal activities through endpoint plating of these cultures for plaque-forming units (PFUs) and colony-forming units (CFUs). Results were then evaluated separately via one-step growth experiments and using different *P. aeruginosa* phages. See Appendix A for discussion of colistin and ciprofloxacin MICs, clinical dosages, and pharmacology. See Appendix B for a detailed description of lysis-profile analysis.

### 2.1. Colistin: High Levels of Anti-Phage Antagonism

**Figure 2:**
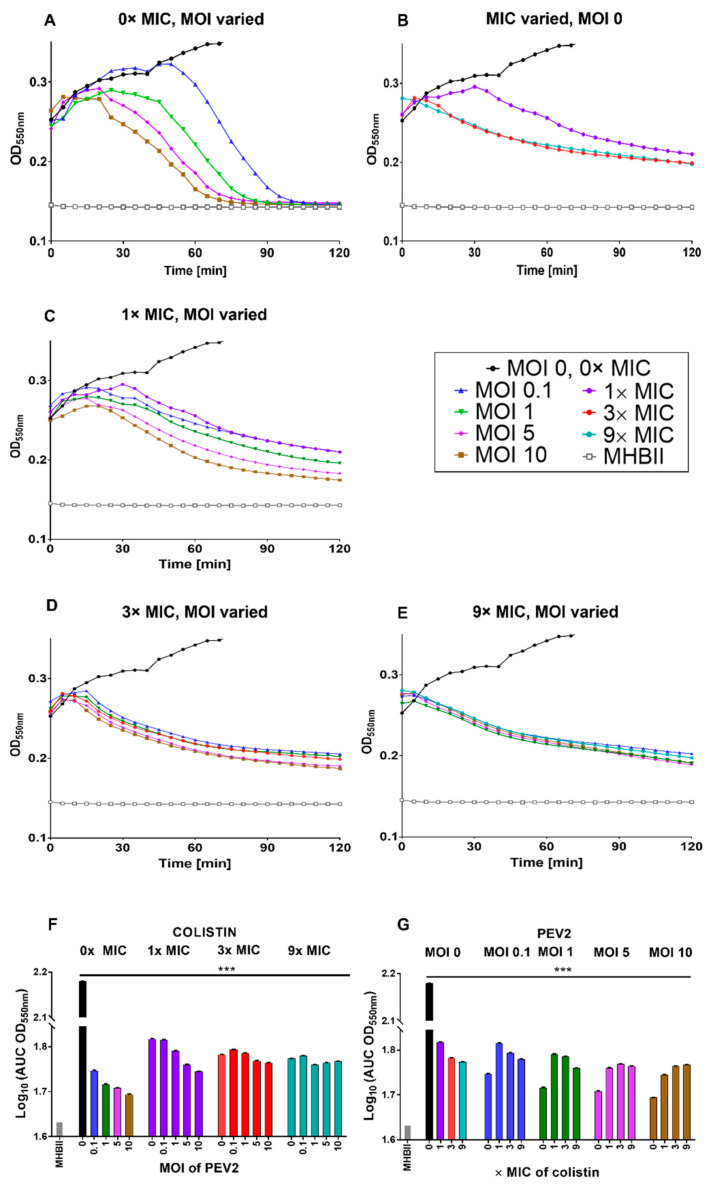
Colistin strongly antagonizes phage PEV2 infection of *P. aeruginosa*. (**A**) Phage PEV2 behavior in the absence of colistin and impact on *P. aeruginosa* PAO1 cultures. (**B**) Impact of different concentrations of colistin on *P. aeruginosa* PAO1 culture without phage. (**C**–**E**) Impacts of 1×, 3×, and 9× MIC concentrations of colistin, respectively, at various MOIs of phage PEV2 including no-phage, colistin-only controls. (**F**,**G**) Area under the curve analysis of PEV2 infection with colistin as derived from lysis profile experiments equivalent to those presented in (**A**–**E**). The smaller the area under a lysis profile curve then the greater the reductions in culture turbidity over time, such as mediated by phage infection. A key is provided in the black frame. Error bars represent standard deviation between samples. See Appendix A for description of the explicit colistin concentrations used and Appendix B for interpretation of lysis profile results. A single representative experiment is shown. [***] *p* < 0.0005, ANOVA.

Colistin (polymyxin class) is a rapid-acting bactericidal antibiotic that disrupts the bacterial outer membrane, leading to leakage of cell contents and bacterial death [85]. This action is achieved partially by colistin displacing the calcium and magnesium bridges that stabilize lipopolysaccharide (LPS) [86,87,88,89,90]. In our study, phage PEV2 lysis profiles in the presence of colistin at 1× MIC showed only limited but nevertheless still clearly detectable phage-associated bacteriolytic activity, as seen with the MOI 5 and 10 curves (Figure 2C and see also Appendix B). No phage-associated bacteriolytic activity appears to occur, however, at higher colistin concentrations (Figure 2D,E). The MOI 0.1 and 1 curves at 1× MIC showed little or no difference relative to no phage addition, suggesting little virion production activity together with low phage-associated bacteriolytic activity (both as seen in Figure 2C). These results are summarized in Figure 2F,G in terms of area under the curves (AUCs) and as heat maps based on log_10_(AUC) calculations (Figure S1A).

Phage PEV2 (family *Podoviridae*) utilizes LPS as its surface receptor (Table S1). This could be a contributor to the observed antagonism of this phage’s infection activity by colistin, as LPS is directly disrupted by that antibiotic. Similar anti-phage antagonism was observed, however, with the infection activity of other *P. aeruginosa* phages, *Autographiviridae* family phages LUZ19 and ΦKMV, that utilize instead type IV pili as their surface receptor (Table S1 and Figures S2 and S3). In adsorption experiments, we did observe phage virion-attachment antagonism in the presence of colistin, even at low concentration (1× MIC), though not to a sufficient degree to indicate a blockage of phage PEV2 nor phage LUZ19 infection abilities, e.g., less than one-half reductions in adsorption rate constants. Though these results are suggestive of a general trend of phage infection activity antagonism by colistin, they are not indicative of the specific cause of that antagonism. We also confirmed [91] that neither colistin nor ciprofloxacin impact virion stability (Figure S5).

### 2.2. Ciprofloxacin: Low Levels of Anti-Phage Antagonism

**Figure 3:**
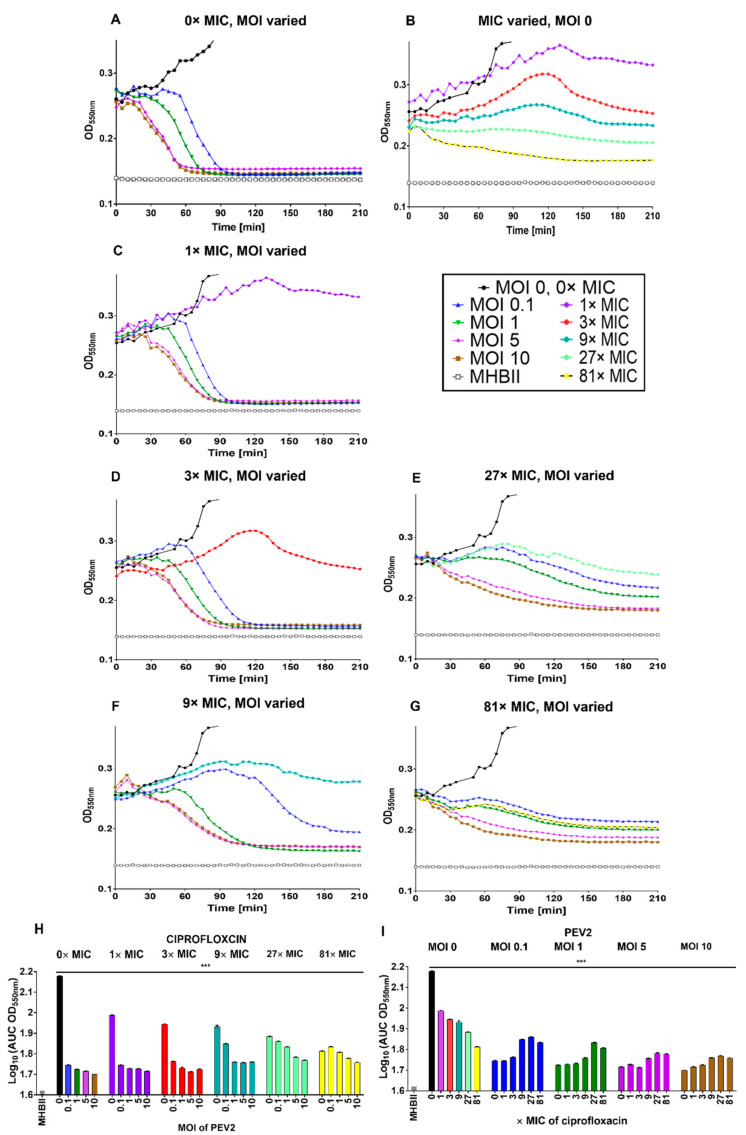
Ciprofloxacin low antagonism of phage PEV2 infection of *P. aeruginosa*. (**A**) Phage PEV2 behavior in the absence of ciprofloxacin and impact on *P. aeruginosa* PAO1 cultures. (**B**) Impact of different concentrations of ciprofloxacin on *P. aeruginosa* PAO1 cultures without phage. (**C**–**G**) Impacts of 1×, 3×, 9×, 27× and 81× MIC concentrations of ciprofloxacin, respectively, at various phage PEV2 MOIs including no-phage ciprofloxacin controls. (**H**,**I**) Area under the curve analysis of PEV2 infection with ciprofloxacin as derived from lysis-profile experiments equivalent to those presented in (**A**–**G**). A key is provided in the black frame. Error bars represent standard deviation between samples. See Appendix A for explicit ciprofloxacin concentrations used and Appendix B for interpretation of lysis profile results. A single representative experiment is shown. [***] *p* < 0.0005, ANOVA. These experiments are indicated in this figure over a longer time scale than that used in Figure 2 due to the greater variation between curves seen later in infections with ciprofloxacin vs. colistin treatments.

Ciprofloxacin, a fluoroquinolone class antibiotic, binds to DNA gyrase (topoisomerase II) or topoisomerase IV in the presence of DNA [92] and prevents the unlinking or decatenation of DNA following its replication while also negatively impacting positive supercoil relaxation and torsional stress relief ahead of transcription and replication complexes [93,94]. Representative lysis profile experiments illustrating the impact of ciprofloxacin on phage PEV2 infection are presented in Figure 3. Contrasting the colistin results (Figure 2), negative impacts on lysis-profile kinetics are minimal at 1× and 3× MIC ciprofloxacin (compare Figure 3A,C,D). At 9× MIC, which is slightly above the maximum serum concentration associated with a 500 mg ciprofloxacin dose in the clinic (Appendix A), bacteriolytic activity also is relatively unchanged, as seen at MOIs of 1, 5, and 10 (Figure 3E). With MOI 0.1 and 9× MIC, however, culture-wide lysis is delayed (Figure 3F), suggesting reduced burst sizes at that ciprofloxacin concentration. See Appendix B for a discussion of these inferences.

At 27× and 81× MIC (8.24 µg/mL and 24.72 µg/mL, respectively), which are well above maximum serum concentration of the highest ciprofloxacin clinical dose (1000 mg, 5.4 µg/mL maximum serum concentration, see Appendix A), some phage bacteriolytic activity might still persist (Figure 3F,G), and perhaps even some virion production (at 27× MIC only). See Figure 3H,I for a summary of ciprofloxacin results in terms of AUCs and Figure S1B for heat map based on log_10_(AUC) calculations. Similar results were obtained for phage LUZ19 and ΦKMV (Figures S6 and S7). We also found no difference in any of these results when phage addition was not concurrent with antibiotic addition but instead delayed by 30 min, suggesting that the relatively low antagonistic impact of ciprofloxacin on phage infection activity is not a consequence of delays in the initiation of ciprofloxacin activity (data not presented).

In adsorption experiments, ciprofloxacin, in contrast to colistin, had no negative impact on phage adsorption rates even at high concentrations (81× MIC) (Figure S4).

### 2.3. Plaque-Forming Unit Counts Support Lysis Profile Results

While virion production is expected during infections of bacteria by strictly lytic phages, it is not a given that this will occur in the presence of antibiotics. Therefore, at the completion of lysis profile-type experiments (Figure 2 and Figure 3), we directly assessed endpoint phage viable counts (PFU).

**Figure 4:**
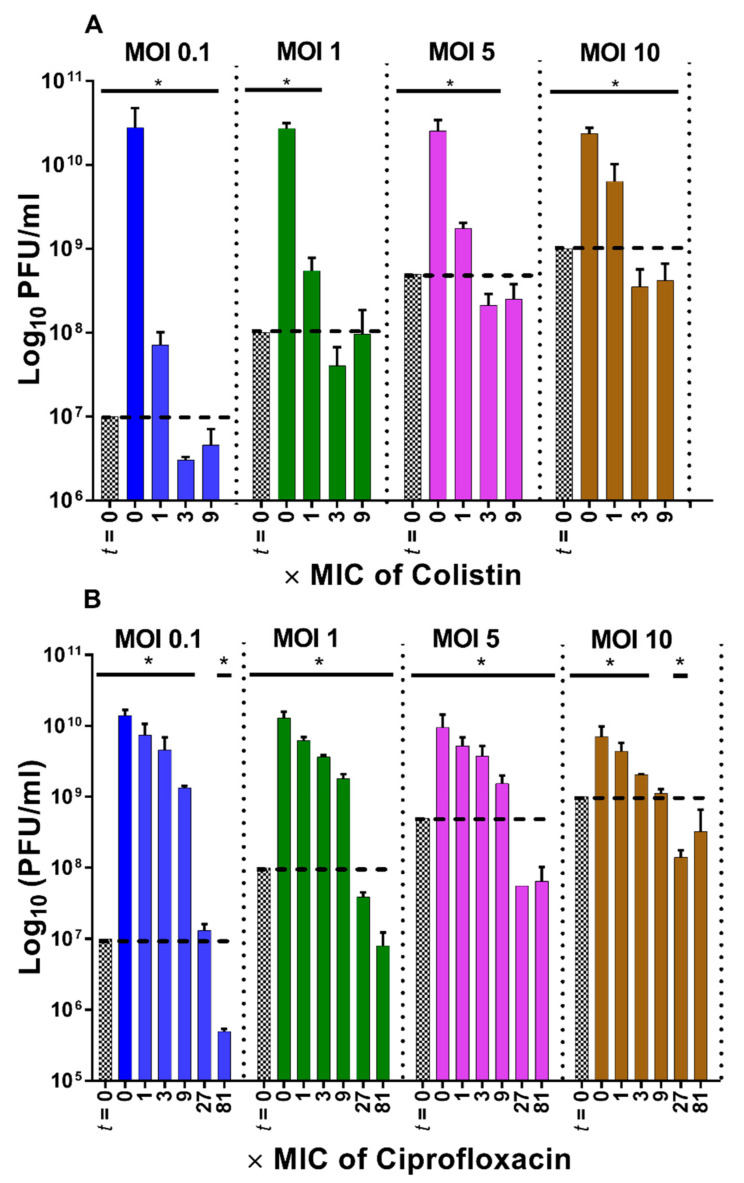
Corroboration of high antagonism of colistin and lower antagonism of ciprofloxacin on phage PEV2 production. Following lysis-profile experiments, counts of phage plaque-forming units (PFUs) were determined in the absence or presence of colistin (**A**) and ciprofloxacin (**B**) treatments (different MICs, see bottoms of graphs) and with different starting MOIs (tops of graphs). PFUs above the dashed lines indicate more phages present than at the start of experiments. Patterned bars (*t* = 0) represent phage titers applied at the beginning of experiments and are compared with 5 h time points (a sampled from cultures immediately following lysis-profile determinations). [*] *p* < 0.05, ANOVA when compared to initial (input) titer (*t* = 0).

Consistent with lysis-profile results (Figure 2), endpoint PFU counts are substantially decreased at 1× MIC colistin in comparison to the no-antibiotic control (over two-log reductions for MOI 0.1, *p* < 0.05), and reduced below input PFUs at 3× MIC or greater (*p* < 0.005, Figure 4A). Also consistent with the lysis profile results, ciprofloxacin at 1× and 3× MIC only minimally decreased phage PEV2 PFUs at all MOIs relative to the no-ciprofloxacin control (*p* < 0.05, Figure 4B). Ciprofloxacin concentrations of 9× MIC, however, reduced phage PEV2 PFU counts by ~1 log at all MOIs tested (*p* < 0.05) relative to the no-ciprofloxacin control and these levels were not significantly different from input PFUs with MOI 10 (Figure 4B). At ciprofloxacin concentrations of 27× and 81× MIC, which are above maximum serum concentration of the highest clinical ciprofloxacin dose (Appendix A), drops in phage titers were observed in comparison to input concentrations of phage PEV2 for MOIs 1 through 10 (*p* < 0.05), but with no change for 27× MIC and MOI 0.1. Together these results are suggestive that PFU determinations can serve as facile means of corroborating lysis-profile results, though at a cost of additional time and effort relative to performing lysis-profile experiments alone.

### 2.4. Colony-Forming Unit Reductions in the Presence of Phages and Antibiotics

**Figure 5:**
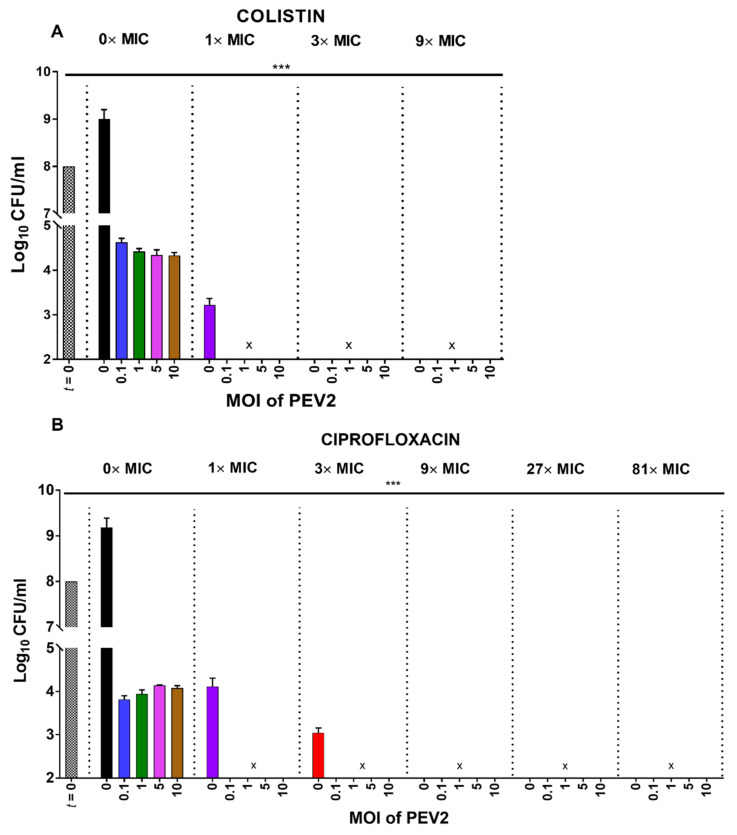
Higher impact of phage-antibiotic combination treatments. Following lysis-profile experiments, counts of bacterial colony-forming units (CFUs) were determined with and without phage treatments (different MOIs, see bottoms of graphs), with and without antibiotic treatments, colistin (**A**) vs. ciprofloxacin (**B**) (different MICs, see tops of graphs), and combined treatments. To prevent bactericidal phage adsorption during enumeration, CFU analyses were performed in the presence of free phage-inactivating virucide consisting of 7.5% black tea infusion and 0.53% FeSO_4_ [95]. [***] *p* < 0.005, ANOVA. [x] represent results below detection limit of this assay (10^2^ CFUs/mL).

Decreases in numbers of CFUs relative to the start of lysis-profile experiments are directly indicative of treatment bactericidal activities. Phage PEV2 reduced CFU counts over the 5 h incubation by at least 99.999% (4 log) in all single (phage-only) and mixed treatments (phage plus antibiotic; *p* < 0.005) (Figure 5). Colistin treatment alone at 1× MIC reduced CFUs over one log more than all phage-only treatments (*p* < 0.005), i.e., about 5 logs. Combination treatments of colistin (for all MICs) and phage PEV2 (for all MOIs) as well as colistin alone, at 3× MIC and greater, were the most successful at reducing CFU counts, by a total of at least 6 logs. There was no significant difference between all phage treatments alone (MOIs 0.1 through 10) and ciprofloxacin treatment alone at 1× MIC. At 3× MIC, ciprofloxacin alone reduced CFU numbers to a greater extent (over 1 log more, *p* < 0.05) than phage-alone treatments. As also in the case of colistin, co-treatment with phages (all MOIs) and ciprofloxacin (all MICs) were the most successful at reducing CFU counts (≥6 log reduction).

Notwithstanding these substantial reductions in CFUs, both with and without phages or antibiotics present (Figure 5), these results do not appear to supply much of a means of distinguishing the impact of colistin vs. ciprofloxacin on phage PEV2 infection activity, and this likely is mostly due to the impact of antibiotic treatment alone reducing CFUs to near or below detection limits. The Figure 5 results, however, are still broadly consistent with those found in Figure 4 in terms of PFU increases. Specifically, in the presence of 1× MIC for both colistin and ciprofloxacin, and 3× MIC for ciprofloxacin, phages were still able to increase in number from nearly to well over 1 log over the course of lysis-profile experiments (Figure 4). At those same MICs, phages reduced CFUs beyond what was attained by antibiotics alone, no matter the starting MOI (Figure 5). This means that even with a starting MOI of 0.1, and certainly with higher starting MOIs, the number of phages present at the end of lysis-profile experiments (Figure 4) should have been sufficient to result in an observable reduction in CFUs relative to antibiotic treatment alone, as indeed was observed (Figure 5). Especially, a final titer of nearly 10^8^ PFU/mL, which is the minimum number of phages in the presence of those MICs, should easily result in a 1-log reduction in bacterial numbers over the course of experiments. Overall, we find that PFU determinations (Figure 5) may provide a better indication especially of low levels of phage replication in the presence of antibiotics than lysis-profile determinations alone (compare Figure 5A with Figure 2C). Furthermore, endpoint CFU determinations alone, as performed here, seem to only be confirmatory that phages along with bactericidal antibiotics are able to kill bacteria.

### 2.5. One-Step Growth Experiments Corroborate Lysis Profile Results

For ~80 years the standard method of assessing phage infection characteristics, particularly determining phage burst sizes and latent period lengths, has been using one-step growth experiments [96,97,98]. These differ from lysis profiles both in terms of the potential for lysis-released virions to adsorb already phage infected bacteria (high potential with lysis profiles vs. low potential with one-step growth experiments) and the means of detection of phage infection activities (culture turbidity for lysis profiles vs. plaque counts for one-step growth experiments). Importantly, however, one-step growth analysis can be much more complicated and time-consuming (e.g., weeks for optimization to specific phages, bacteria, and conditions) and are not easily automated. The lysis-profile analyses presented here, i.e., Figure 2 and Figure 3, by contrast, involve little optimization, require only a few hours to set up and run, use standard incubating and shaking microtiter plate reading machines, and like other phage assays based on optical density readings [99], can be performed with high throughput.

Given the importance of one-step growth analysis as the standard means of assessing phage infection activities, we have employed it to test the accuracy of the presented lysis profiles (Figure 2 and Figure 3) as predictors of these same activities. In the absence of antibiotics, we found that the phage PEV2 latent period lasts ~18 min, resulting in a burst size of 135 ± 5.6 new virions. This timing of lysis seems to correspond, as expected, more or less to the MOI 5 and 10 lysis profiles shown in Figure 2A and Figure 3A.

**Figure 6:**
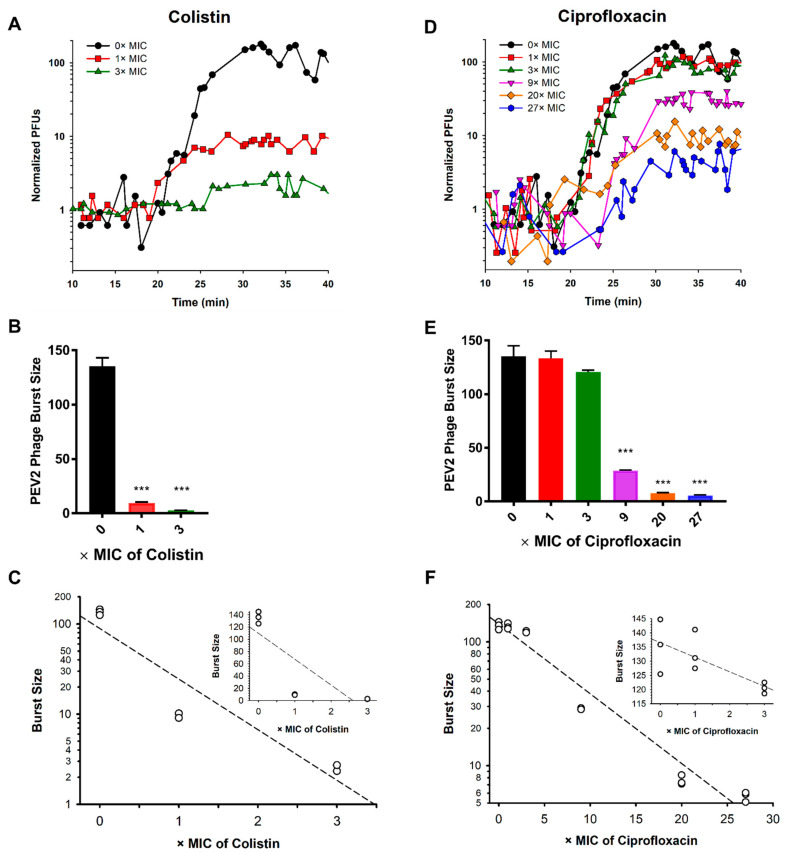
PEV2 one-step infection cycle characteristics in the absence or presence of antibiotic. Shown are one-step growth curves of phage PEV2 infection (MOI 0.05) in the absence or presence of either colistin (**A**) or ciprofloxacin (**D**), in both cases with numbers of PFUs normalized so that the average initial numbers of phage-infected bacteria for all curves are set to 1.0. Graphed in the second row are phage PEV2 burst sizes determined in the absence or presence of different colistin (**B**) or ciprofloxacin (**E**) concentrations. Error bars represent standard deviations between three experiments (biological repeats). Shown in the bottom row is the relationship between burst size and either colistin (panel (**C**), m = −0.561, r = 0.938) or ciprofloxacin (panel (**F**), m = −0.056, r = 0.982) concentrations. Inserts show burst sizes using linear scales at the first three concentrations (0×, 1×, and 3× MIC). Mean values of burst size are calculated based on 3 separate experiments. The dotted line represents a trend line. [***] *p* < 0.005, ANOVA, (r) linear regression, (m) slope. See Appendix A for detailed antibiotics concentrations.

At 1× and 3× MIC, colistin did not have a significant impact on the phage PEV2 latent period length (Figure 6A), a result that was not detectable in our lysis profile assays (Figure 2C,D). At higher colistin concentrations (>3× MIC), lawn growth during PFU assessment was inhibited, presumably by antibiotic carry over, and therefore one-step growth assessment could not be performed. Contrasting the consistency of latent periods seen in the presence of 1× and 3× MIC colistin, burst sizes were substantially reduced (*p* < 0.005), to 9.6 ± 0.57 (reduction of 92.9%) and 2.5 ± 0.2 (reduction of 98.1%), respectively, given phage infection exposure to these concentrations of colistin (Figure 6B,C).

The phage PEV2 latent period length did not change with 1× or 3× MICs but was delayed at 9× MIC and higher of ciprofloxacin, as mirrored by the MOI 5 and 10 lysis profile results presented in Figure 3. Burst sizes were 133.2 ± 4.1, 120.5 ± 1.1, 28.8 ± 0.3 phage PFUs per infected bacterial cell, corresponding to 1.5%, 10.9% and 78.7% reductions, respectively (Figure 6E,F). When ciprofloxacin concentrations were increased to 20× and 27× MIC, the phage PEV2 latent period was postponed by ~3 and ~7 min, respectively (Figure 6D), and burst sizes dropped to 7.6 ± 0.4 and 5.7 ± 0.3 phages/infected bacterium (Figure 6E,F), the latter corresponding to reductions of 94.4% and 95.8%. Burst size changes during lysis profiles should be most detectable with MOI 0.1 curves and seen as delays in optical density reductions as phage populations more slowly grow to multiplicities in excess of 1, particularly as bacterial numbers grow as well due to binary fission prior to their becoming phage infected. Such a delay in culture-wide lysis with MOI 0.1 is most obviously seen with 9× MIC ciprofloxacin in Figure 3F.

In Figure 6C,F, the rate of decline of phage PEV2 burst sizes as functions of colistin and ciprofloxacin concentrations are indicated. Based on the calculated slopes of the log-linear graphs (main graphs, not insets), we can conclude that increasing colistin concentrations reduce the phage PEV2 burst size at a rate that is ten-fold greater than the corresponding ciprofloxacin antagonistic impact in terms of MICs: (*m* = −0.561)/(*m* = −0.056) = 10.

These one-step growth results are consistent with the lysis profile and PFU results presented in Figure 2, Figure 3 and Figure 4, indicating for example that ciprofloxacin does not excessively interfere with phage infection activities until relatively high antibiotic concentrations (>9× MIC) vs. colistin which substantially interferes with these phage activities even at relatively low concentrations (1× MIC). Both bacteriolysis and virion production seem to not be completely interfered with during lysis profiles at 27× MIC of ciprofloxacin (Figure 3E, MOIs 5 or 10, and Figure 4B, MOI 0.1, respectively) nor bacteriolysis with 1× MIC of colistin (Figure 2C), though both are at or nearing the limits of detection in each case. These results would appear to be consistent with the observed retention in one-step growth experiments of low but still present burst sizes in the presence of those concentrations of the respective antibiotics.

## 3. Discussion

In combination therapies, two or more agents possessing different mechanisms of action can enhance each other’s activities [100,101,102], broaden activity spectra [101,103,104,105], reduce the potential for resistance evolution [106,107,108,109,110,111,112,113], and improve penetration into biofilms [114]. They can, however, also be antagonistic to each other’s functioning [16,17,115]. Antibiotics, for example, are known to be potentially antagonistic to phage infection activities [47]. One common aspect of both pre-clinical [34,42,47,102,114,116,117,118,119,120,121,122] and clinical phage therapy treatments [26,34,35,36,37,38,39,40,41,42,43,44,45,46,47,48,49,50,51,52,53,54,55,56,57,58,59,60,61,62,63,64,65,66,67,68,69,70,71,72,73,74], however, is concurrent combination of phages with antibiotics. Here we have assessed the potential of two different antibiotics, colistin (polymyxin) and ciprofloxacin (fluoroquinolone), to interfere with the infection activities of *P. aeruginosa* phage PEV2, LUZ19, and ΦKMV at bacterial growth-inhibiting antibiotic concentrations (greater than or equal to MIC). We found that colistin was far more antagonistic than ciprofloxacin, with a ten-fold greater rate of reduction in burst size seen as a function of increasing colistin concentrations vs. increasing ciprofloxacin concentrations.

As follows, we consider the extent to which antagonistic impacts on phage infection activities stemming from the action of these two antibiotics have been observed in other studies. We note as a general comment, however, that it is possible for many studies that MIC determinations and phage experiments were conducted using different media, perhaps resulting in different actual MICs under experimental conditions from those reported. We therefore have indicated what media phage experiments were performed in along with MIC determinations, which we assume as a default were likely performed using Mueller-Hinton media. By contrast, all experiments presented in the current study were conducted using exclusively Mueller-Hinton Broth, cation-adjusted.

### 3.1. High Antagonistic Impact of Colistin

In this section, we consider studies that have explored the impact of colistin on the infection activity of various phages. David et al. [91], for instance, showed that colistin at 1× MIC (1 µg/mL) inhibited phage production during infection of *Mycobacterium aurum* in heart-infusion broth. Often, though, this information must be inferred rather than having been measured directly, including whether or not antibiotic concentrations used were sufficient to inhibit bacterial growth or metabolism. Jansen et al. [42], for example, reported phage-mediated reductions of *Acinetobacter baumannii* culture growth in lysogeny broth in the presence of 1× and 4× MIC colistin (0.5 to 8 mg/L), though not at 2× MIC colistin. They also observed, however, little reduction in culture growth in the presence of these concentrations of colistin alone.

Using a cocktail of two phages, Chaudhry et al. [102] similarly observed substantial phage population growth as well as phage-associated reductions in *P. aeruginosa* PA14 (vs. PAO1 as used here) with 8× MIC (20 µg/mL) colistin suspended in lysogeny broth. In these experiments, however, phages were targeting bacterial biofilms rather than planktonic bacteria, and biofilms tend to be more tolerant of colistin than bacteria found in broth [123]. MICs do not appear to have been determined in this study using biofilm-grown bacteria, however, and neither 1× nor 8× MIC colistin alone had much negative impact on so-treated biofilms. With similar caveats, Danis-Wlodarczyk et al. [124] observed in tryptic-soy broth that application of the giant *phiKZvirus* KTN4 (*P. aeruginosa* phage), together with 100 µM (116 µg/mL) of colistin, substantially reduced a *P. aeruginosa* PAO1 72 h old biofilm, while colistin alone did not have an impact and phage-alone treatment reduced biofilm to a lesser extent than the combination treatment. The phage, however, was not more effective than colistin alone against 24 h or 48 h biofilm. MIC was not determined, but the colistin concentration used was found to reduce the growth of 24 h biofilm, leaving open a possibility that it was less effective against the 72 h biofilm.

Overall, then, evidence of substantial phage replication while infecting bacteria that are truly colistin-inhibited in their growth is not robust. Carried out under conditions resembling those employed for MIC determinations (MHB), we have found that colistin is strongly antagonistic toward phage PEV2 bacteriolytic and virion production activities, e.g., with greater than 90% reduction in burst size at 1× MIC.

### 3.2. Low Antagonism of Ciprofloxacin

Consistent with our in vitro findings is the in vivo study by Oechslin et al. [125]. They found synergistic impacts of ciprofloxacin and a phage cocktail on experimental *P. aeruginosa* endocarditis in rats. This is notable since ciprofloxacin alone, at the same dosage, displayed substantial antibacterial activities in this in vivo system and thus presumably exceeded MIC there. Using a tryptic soy broth model, Oechslin et al. also found that numbers of *P. aeruginosa* cells were substantially reduced (~6 logs) after 6 h of exposure to concurrent co-treatments of a phage cocktail (MOI 1, 10^8^ PFUs/mL) and ciprofloxacin (2.5× MIC, 0.475 µg/mL) as well as by phage-only treatment, versus only an inhibition of bacterial growth when treated with ciprofloxacin alone. This result is suggestive of phage virion production in both the presence and absence of 2.5× MIC ciprofloxacin since the starting MOI of 1 alone at best should kill only 63% of the bacteria present [126], though we are uncertain how MIC was determined in this study.

Here, of course, we have also shown that phage infection activities can persist even at substantially higher multiples of ciprofloxacin MIC, with persistence of phage production activity at even ten times 2.5× MIC and under conditions that better match those of standard, broth-based MIC determinations. Otherwise, we have found it difficult to find evidence from the literature that phage replication can occur in the presence of truly inhibitory concentrations of ciprofloxacin. To clarify the latter point, see Appendix C where we review this literature.

### 3.3. Conclusions

Unlike most other studies that have found some compatibility between ciprofloxacin and phage treatments, here we measured antagonism under conditions that are equivalent to determinations of MICs, including the use of planktonic bacteria and Mueller-Hinton broth. We found that ciprofloxacin could be well suited for concurrent antibiotic-phage therapy co-treatment vs. colistin. This assertion is based on phage PEV2 displaying substantial bactericidal, bacteriolytic, and phage production activities even at clinically high levels ciprofloxacin, as corroborated with phages ΦKMV and LUZ19, though it will be important to test in the future additional phages for this property before progressing, e.g., to animal studies. It is striking, though, that explorations of antagonism of phage infection activity is rarely attempted before progressing even into the clinic as we have found that roughly two-thirds of clinical phage therapy studies published since 2000 employ concurrent phage-antibiotic combination therapies. Methodologically, we thus advance lysis profiles as a rapid and inexpensive broth-based assay for screening for antibiotic antagonistic impacts on phage infection activities given consistency between observations made using lysis profiles and standard one-step growth assays.

## 4. Materials and Methods

### 4.1. Bacteria, Antibiotics, and Phages

*P. aeruginosa* PAO1 Krylov is used as a reference strain in this study, as provided by Jean-Paul Pirnay (Queen Astrid Military Hospital, Brussels, Belgium). *P. aeruginosa* surface mutants used for phage receptor analysis are described in Table S1 and are isogenic to the *P. aeruginosa* PAO1 strain from Harvard University. Mueller-Hinton Broth II (MHB), cation-adjusted (Becton Dickinson, NJ, USA), is used for bacteria liquid cultures, supplemented where indicated with different concentrations of ciprofloxacin (ciprofloxacin hydrochloride, usp reference standard, USA) or colistin (colistin sulfate, usp reference standard, USA). Minimal inhibitory concentrations (MICs) were measured by exposing a broth bacterial culture to increasing concentrations of antibiotic in MHB, as previously described [115]. MIC for ciprofloxacin was determined as 0.91 µM = 303.2 ng/mL, and for colistin MIC was found to be 39.06 µM = 68.75 µg/mL (see Appendix A for further discussion). Antibiotic concentrations in basic experiments were varied as 0× (no antibiotic), 1×, 3×, 9×, 27×, and 81× MIC (× = times). Additional antibiotic concentrations were tested for ciprofloxacin between 9× and 27× MIC toward ascertaining maximal antibiotic concentrations that will still allow for phage bacteriolytic and virion production activities. We also confirmed that *P. aeruginosa* PA01 failed to replicate at 1× MIC colistin and ciprofloxacin using microscopic total count determinations (data not shown).

*P. aeruginosa* phage PEV2 (NC_031063.1, family *Podoviridae*, subfamily *Sepvirinae*, genus *Litunavirus*) was kindly provided by Elizabeth Kutter (Evergreen State College, Olympia, WA, USA). *P. aeruginosa* phages LUZ19 (NC_010326.1) and ΦKMV (NC_005045.1) (family *Autographiviridae*, subfamily *Krylovirinae*, genus *Phikmvvirus*) were kindly provided by Rob Lavigne (KU Leuven, Leuven, Belgium). Phages were propagated in LB (Lysogeny Broth, Fisher Scientific, Hampton, NH, USA), purified via PEG precipitation (25% polyethylene glycol 8000, VWR, Radnor, PA, USA), subsequently suspended in phage buffer (10 mM Tris-HCl, Sigma,10 mM MgSO_4_, 150 mM NaCl, pH 7.5, Fisher Scientific), and stored at 4 °C, as previously described [127]. Phage titers were assessed using the double-agar layer method [128,129] and defined as plaque-forming units per ml (PFU/mL).

Bacterial densities were estimated as colony-forming units per ml (CFU/mL). When CFUs were determined under experimental conditions, e.g., as in the presence of phages, cultures were first exposed, prior to plating, to virucide consisting of 7.5% black tea infusion (Ceylon tea from Sri Lanka, Ahmad Tea, UK) and 0.53% FeSO_4_ (Sigma, Germany), prepared according to the Chibeu and Balamurugan [95] protocol. Tea type was chosen based on the results of de Siqueira et al. [130]. The virucide was used to inactive phage virions to avoid overestimation of phage-mediated bacterial killing (bactericidal activity) prior to plating. In separate experiments, this treatment was found not to affect bacterial viable counts (data not presented).

### 4.2. Phage Surface Receptor Analysis

Phage specificity to a particular bacterial receptor was tested on *P. aeruginosa* PAO1 mutants deficient in biosynthesis of flagella, type IV pili, alginate production, or structure of their lipopolysaccharide (LPS) was modified (Table S1), as previously described [124]. Bacterial susceptibility to phage was identified by spot testing (10 μL volume) using a suspension of 10^7^ PFU/mL. The plates were checked after 4–6 h and again after 18 h for the presence of a spot corresponding to confluent lysis located beneath applied volumes.

### 4.3. Analysis of Virion Attachment Antagonism

The impact of ciprofloxacin and colistin on phage virion attachment to bacterial surface was tested with a standard adsorption curve analysis [131]. In short, the PAO1 strain was mixed with PEV2 or LUZ19 phage at MOI 0.01 in MHBII with or without the presence of antibiotics, colistin, or ciprofloxacin (concertation range between 1× and 81× MIC). Immediately after mixing and further at 30 s intervals, 100 µL aliquots were taken and mixed with 850 µL of MHB supplemented with 50 µL of chloroform. These mixtures were incubated for 10 min to kill any remaining phage-infected bacteria, centrifuged (10 min, 14,000 rpm) and phage titers were established from the supernatant by double agar layer method [128] to determine the amount of non-adsorbed or reversibly adsorbed phage.

### 4.4. Phage Stability in the Presence of Antibiotics

Phage stability in the presence of antibiotics was assessed by mixing phages PEV2, LUZ19 or ΦKMV (1 × 10^8^ pfu/mL) and antibiotics, colistin or ciprofloxacin (concertation range between 1× and 81× MIC) in 1 mL of MHB. As a negative control, we used a mixture of phage and phage buffer or MHB. As a positive control, we used a mixture of phage and 0.1% SDS solution [132,133]. Samples were further incubated at 37 °C for 24 h and subsequently phage titers were evaluated with the use of the standard double agar method [128].

### 4.5. Analysis of Phage Infection Activity via Lysis Profiles

Lysis profiles involve the addition of phages to relatively high densities of bacteria, with phage impact on bacteria observed as reductions in bacterial culture turbidity. To assure a robust measure of culture turbidity declines, i.e., as is generally assumed in these experiments to be associated with phage-induced bacterial lysis, cultures are not diluted following phage adsorption (contrast to one-step growth experiments, as described in the following section). The turbidity measurements were conducted at OD_550nm_ with the use of SpectraMax i3x Multi-mode Microplate Reader (Molecular Devices, San Jose, CA, USA).

Phage, antibiotic, or both were added to log-phase *P. aeruginosa* PAO1 (OD_600nm_ = 0.32, ~1.1 × 10^8^ CFU/mL; time, *t* = 0 min). Subsequent incubations were at least 2 h in length and took place as 200 µL/well volumes within 96-well microtiter plates. For incubations, these plates were covered with Breathe-Easy Film (Diversified Biotech, Dedham, MA, USA) and kept at 37 °C. Turbidity measurements were taken every 5 min and cultures were shaken for 3 sec before every read. All materials including microtiter plates were preincubated at experimental temperatures to limit physiological stress, and phages as well as antibiotics were prepared in MHB prior to use. Negative controls consisted of untreated PAO1 and MHB only. Input multiplicities of infection (MOIs) [134,135] ranged from 0.1 (for virion production assessment) to 10 (for phage-induced bacteriolysis assessment). These MOIs corresponded to within-well titers of ~1 × 10^7^, ~1 × 10^8^, ~5.0 × 10^8^, and 1 × 10^9^ PFU/mL (or MOIs of 0.1, 1, 5, and 10, respectively). Following lysis profile determinations, PFU and CFU counts were assessed, initially via spot test assays, and for PFUs further via the double-layer method [128,129]. Experiments were performed in three technical repeats and three biological repeats.

### 4.6. Analysis of Phage Infection Activity via One-Step Growth

One-step growth experiments were undertaken according to previously established methods [96,97,98,136], with modifications. A volume of 900 µL of a mid-exponential bacterial culture (final concentration ~2 × 10^8^ CFU/mL) in MHB was mixed with 100 µL of PEV2 phage suspension (final concentration ~1.0 × 10^6^ PFU/mL) to obtain an input MOI of 0.005. Phages were allowed to adsorb for 8 min at 37 °C, after which time the mixture is diluted 10^4^-fold, with samples then taken at 20–30 s intervals for titer determination. PFU/mL titers presented in the graphs are values from which unabsorbed phages values were subtracted. Antibiotic, if present, was added to PAO1 cultures 15 min prior to phage addition as well as to all dilution media. At least three biological repeats were performed.

### 4.7. Statistics

The area under the curve (AUC) was calculated by GraphPad Prism version 7.00 for Windows (GraphPad Software, La Jolla, CA, USA, www.graphpad.com, accessed on 15 October 2021) based on 5 h lysis profiles. Statistics were performed with a one-way ANOVA test. Statistical software R [137] was used for statistical computing and graphics based on log_10_(AUC) values. Heat maps were plotted with pheatmap [138]. The pvclust [139] was used for assessing the uncertainty in hierarchical cluster analysis. For each cluster in hierarchical clustering, probability values (*p*-values) are calculated via multiscale bootstrap resampling and ranging between 0 and 1, which indicates how strong the cluster is supported by data. Two types of *p*-values are available: approximately unbiased (AU) *p*-value and bootstrap probability (BP) value. The AU *p*-value, which is calculated by multiscale bootstrap resampling, is a superior approximation to unbiased *p*-value over BP value computed by normal bootstrap resampling. With pvclust, hierarchical cluster analysis is performed via function hclust and automatically computes *p*-values for all clusters contained in the clustering of original data.

## Figures and Tables

**Figure A1:**
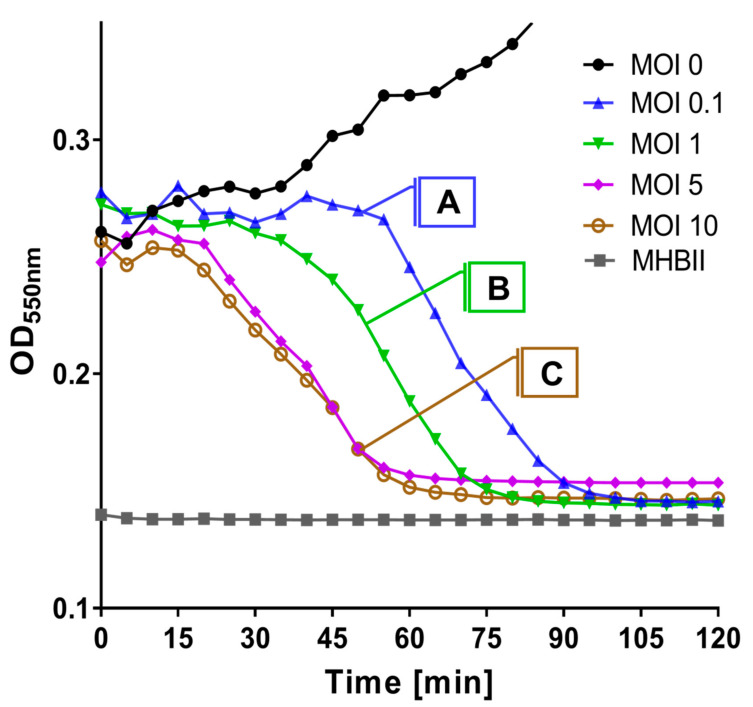
Phage PEV2 lysis profiles. (Curve A) Low starting MOI (=0.1): Phages must replicate and produce new virions to give rise to culture-wide bacterial lysis, as will occur after a minimum of 2 infection cycles. (Curve B) Intermediate MOI (=1): Culture-wide bacterial lysis is unlikely to be due to lysis from without (an extracellularly, enzymatically induced lysis of bacterial cells following high-multiplicity phage adsorptions), though lysis may be delayed relative to curve C due to delayed and only fractional virion adsorption. (Curve C) High starting MOI (5 or 10): Timing of bacterial lysis should occur after a single phage latent period, unless somewhat sooner due to lysis from without. For especially MOIs 5 or 10, phage infections need only retain bacteriolytic activity to result in robust drops in turbidity.

## Data Availability

The data presented in this study are available in article.

## Supplementary Materials

The following are available online at https://www.mdpi.com/article/10.3390/ph14111162/s1, Figure S1: Heat map analyses of PEV2 infection with colistin (A) and ciprofloxacin (B), Figure S2: High antagonism of colistin on LUZ19 infection activities, Figure S3: High antagonism of colistin on ΦKMV infection activities, Figure S4: Phage adsorption curves reveals high antagonism of colistin, Figure S5: Phage stability in the presence of colistin and ciprofloxacin, Figure S6: Low antagonism of ciprofloxacin on LUZ19 infection activities, Figure S7: Low antagonism of ciprofloxacin on ΦKMV infection activities, Table S1: Phage receptor identification using isogenic *P. aeruginosa* PAO1 surface mutants.

## Appendix A. Antibiotic MICs, Dosing, and Pharmacology

### Appendix A.1. Colistin

Colistin can be administrated parenterally, intravenously, or via inhalation. Currently there are no clear dosing guidelines, pharmacokinetics, or pharmacodynamics provided by the FDA. In Table A1 we present recommended dosing and target serum concentrations based on the most comprehensive study of colistin treatment of ill patients and recently published International Consensus Guidelines for the Optimal Use of Polymyxins [140].

**Table A1:** Colistin and ciprofloxacin pharmacology.

Antibiotic	Dose	Maximum Serum Concentration	Area Under Curve (AUC)
Colistin (polymyxin E) *	75–410 mg ^2^	0.48–9.38 µg/mL	N.D.
	300–360 mg CBA for a patient with normal renal function ^3^	~2 µg/mL [140,141,142] *	~50 mg × h/mL **
Ciprofloxacin ^1^	250 mg	1.2 µg/mL	4.8 µg × h/mL
	500 mg	2.4 µg/mL	11.6 µg × h/mL
	750 mg	4.3 µg/mL	20.2 µg × h/mL
	1000 mg	5.4 µg/mL	30.8 µg × h/mL

^1^ presented based on FDA dosing analysis and recommendations [143]. ^2^ Garonzik et al. [141]. Their recommendations were against exceeding 300 mg “colistin base activity” per day (about 5 mg/kg/day) for either the loading or maintenance doses. ^3^ Based on International Consensus Guidelines for the Optimal Use of the Polymyxins: Endorsed by the American College of Clinical Pharmacy (ACCP), European Society of Clinical Microbiology and Infectious Diseases (ESCMID), Infectious Diseases Society of America (IDSA), International Society for Antiinfective Pharmacology (ISAP), Society of Critical Care Medicine (SCCM), and Society of Infectious Diseases Pharmacists (SIDP) [140]. CBA: colistin base activity. N.D.: not determined. * the target average steady-state total plasma colistin concentrations (C_ss,avg_) of ≥2.5 mg/L may increase the risk of nephrotoxicity (controversial in the field), which complicates the clinical decision to choose the appropriate colistin dosing [144,145,146]. ** maximum tolerable exposure.

Since there are no clear guidelines for colistin treatment, in our study we performed first a standard broth-based MIC analysis with the *P. aeruginosa* PAO1 reference strain (1× MIC = 39.06 µM = 68.75 µg/mL) and we employed the following concentrations in further experiments: 1× MIC = 39.06 µM = 68.75 µg/mL, 3× MIC = 117.18 µM = 206.25 µg/mL, 9× MIC = 351.54 µM = 618.75 µg/mL, 20× MIC = 18.2 µM = 6.1 µg/mL, 27× MIC = 24.70 µM = 8.24 µg/mL, and 81× MIC = 74.10 µM = 24.72 µg/mL.

### Appendix A.2. Ciprofloxacin

In the clinic, ciprofloxacin is administrated in two forms, film-coated tablets—available in 100 mg, 250 mg, 500 mg, and 750 mg strengths—or oral suspension, available in 5% (5 g ciprofloxacin in 100 mL) and 10% (10 g ciprofloxacin in 100 mL) strengths [2]. Ciprofloxacin maximum serum concentrations and AUC in correlation to dose are presented in Table A1, as based on FDA directions for ciprofloxacin dosing and pharmacology [143].

For this study, a standard broth-based MIC analysis was performed, which indicated that ciprofloxacin inhibits macroscopically growth of sensitive *P. aeruginosa* PAO1 Krylov strain at 305.2 pg/mL (0.91 µM). Based on these results and FDA ciprofloxacin dose recommendations, we established concentrations span for further experiments as follows: 1× MIC = 0.91 µM = 303.2 ng/mL, 3× MIC = 2.74 µM = 909.5 ng/mL, 9× MIC = 8.23 µM = 2.73 µg/mL, 20× MIC = 18.3 µM = 6.1 µg/mL, 27× MIC = 24.70 µM = 8.19 µg/mL, and 81× MIC = 74.11 µM = 24.56 µg/mL.

## Appendix B. Interpretation of Lysis Profiles

Phage infection characteristics can be inferred from lysis-profile experiments, with different aspects assessed given different starting (input) phage MOIs. These, as seen with phage PEV2 without antibiotic treatments, are summarized in Figure A1 and below. Specifically, MOIs of 0.1, 1, 5, and 10 have been employed throughout this study, which can provide evidence of virion production, phage-induced bacterial lysis (i.e., lysis from within [80]), and phage lysis timing (latent period length), respectively.

### Appendix B.1. Curve A. Indicating Moderate or Greater Virion Production

Starting with an input MOI of 0.1, the timing of culture-wide lysis should begin after no less than two consecutive phage latent periods rather than after just one (curve A, Figure A1). This is because initially, at this MOI, only about 10% of the bacterial population can become phage infected, and it will take one latent period (the first latent period) before new virions are released. These new virions, if produced in sufficient numbers, can then adsorb and infect additional bacteria. Such ‘two-step’ growth can then result in relatively rapid culture-wide lysis, but only if phage burst sizes are sufficiently large to result in infection of the majority of bacteria present. That is, such as in excess of ~10 phages/infected bacterium so that most of the bacteria in the culture will have become phage infected after the first phage latent period. Observations of timely culture-wide reductions in turbidity at this low MOI thus can serve as an indication of phage virion production such as in the presence of antibiotic.

### Appendix B.2. Curve B. Indicating Lysis from Within

With MOI 1 (curve B, Figure A1) we observe slower lysis than with MOIs 5 or 10 (curve C, Figure A1). This delay could be a consequence of slow initial phage adsorption to bacteria, as with an input MOI of 1 the rate that a bacterium becomes adsorbed by at least one phage virion should be up to five- to ten-fold slower than with the higher input MOIs. In addition, with MOI 1 only approximately one-half [63% = 100 × (1 − e^−1^)] of bacteria will be initially infected, i.e., as due to Poisson distributions of phage adsorption [126]. Slower culture-wide lysis at the end of phage latent periods given a starting MOI of 1 thereby can result both because fewer bacteria are infected and bacteria are infected more slowly relative to cultures initiated with MOIs of 5 or 10. Notwithstanding such delays, culture-wide clearing for MOI 1 curves should be indicative of an occurrence of lysis from within as under these low-multiplicity conditions lysis from without (below) is unlikely.

### Appendix B.3. Curve C. Indicating Timing of Lysis

The timing of lysis in lysis profiles, especially given starting MOIs of somewhat greater than 1 (here MOI 5 or 10), can approximate phage latent periods. This is seen here as the initial drop in turbidity observed soon after 15 min with the curves labeled as curve C in Figure A1. These higher input MOIs under certain circumstances, perhaps especially given antibiotic presence, might instead result from lysis from without. That is, a rapid, external digestion of cells walls and then bacterial lysis following high multiplicity virion adsorption [147]. Contrast instead lysis from within, which is phage-induced bacterial lysis as it normally occurs at the end of a phage’s latent period [148], which with greater certainty is what is observed with given lower MOI experiments (curves A and B, Figure A1).

## Appendix C. Complications in Determining Ciprofloxacin Impact on Phages

A handful of studies have evaluated the impact of 1× MIC or greater ciprofloxacin concentrations on the in vitro infection activities of various phages. These generally have been measured over many rounds of phage infection or with other issues that make it difficult to ascertain the impact of ciprofloxacin, at inhibiting concentrations, on phage infection activities. We review these studies as follows.

Valério et al. [149] observed an absence of an additional reduction in CFUs when *Escherichia coli* was treated in phosphate-buffered saline with phages (about 7 × 10^6^ PFU/mL) and 1× MIC (0.5 µg/mL) ciprofloxacin, vs. antibiotic alone. Without a carbon or energy source, however, our expectation is one of minimal phage replication. Furthermore, this is not a substantial starting titer of phages, and even though it represented a ratio of added phages to bacteria of 100, densities of bacteria likely were insufficient (<10^5^ CFU/mL) to support substantial additional phage population growth. Therefore, it is difficult to conclude whether or not ciprofloxacin in this case was inhibiting phage infection activities. MICs were determined using Mueller-Hinton agar.

Also with *E. coli*, Lopes et al. [150] observed less phage population growth in the presence of 1× MIC (0.25 mg/mL) ciprofloxacin than without, and no increases in phage numbers were seen at 2× MIC (0.5 mg/mL). These experiments were conducted in tryptic soy broth, however, while MICs were determined using MHB.

Alternatively, Jansen et al. [42] observed reductions in bacterial concentrations over antibiotic alone in the presence of an *A. baumannii* host and slightly over 1× MIC (0.125 mg/L) ciprofloxacin, with these experiments carried out in lysogeny broth. Bacterial growth was only slightly reduced in the presence of this amount of ciprofloxacin, however, with an endpoint OD_590_ in the absence of phages reduced from slightly more than 0.9 with no antibiotic to slightly less than 0.8 with 1× MIC. Roughly 0.5× MIC ciprofloxacin instead resulted in an endpoint OD_590_ of slightly more than 0.8, also absent phages. The means of MIC determination does not appear to be reported.

Otherwise are studies carried out in biofilms, which can display greater tolerance to ciprofloxacin activity than broth-growing bacteria [123]. Thus, and just as with colistin (Section 3.1), ciprofloxacin concentrations that are found to be inhibitory of bacterial growth in broth may not be inhibitory to bacterial metabolic activities within biofilms. Working with *P. aeruginosa* PA14 biofilms, Chaudhry et al. [102] thus found that 1× MIC (0.8 µg/mL) ciprofloxacin did not block the production of new virions while 8× MIC (6.4 µg/mL) did, though MIC was determined using planktonic rather than biofilm cultures. Indeed, 1× MIC ciprofloxacin alone had little impact on biofilm presence while 8× MIC ciprofloxacin acting alone had a substantial impact.

Similar results were seen by Dickey et al. [151] with *Staphylococcus aureus* biofilm in MHB and phage-ciprofloxacin co-treatment. Treatment with phage and ciprofloxacin at 2× MIC substantially reduced the number of biofilm-embedded bacteria, while ciprofloxacin alone also at 2× MIC did not reduce CFUs. When antibiotic concentration was increased to 10× MIC, CFUs were reduced, but there was no additional reduction with co-treatment with phages. This correlates with phage production in the presence of 2× MIC ciprofloxacin but no phage production in the presence of 10× MIC. It is uncertain what medium was used to determine MICs but this could have been MHB.

Verma et al. [152] saw no change in phage impact on *Klebsiella pneumoniae* biofilms with and without ciprofloxacin (10 µg/mL, >>1× MIC). As these twelve-hour biofilms appear to have not grown further in the presence of the antibiotic, their result is suggestive of a retention of phage anti-biofilm activity in the presence of ciprofloxacin. The phage used in that study, however, appeared to produce an anti-biofilm depolymerase enzyme, which could have reduced biofilm presence without corresponding phage infection activity, i.e., [153,154,155,156,157,158].

Luscher et al. [159] treated 6 h *P. aeruginosa* PAO1 infections of Calu-3 epithelial cell lines in minimal essential medium with phages in the presence of 16× MIC ciprofloxacin (4.0 μg/mL, which roughly corresponds to a maximum serum concentration of 750 mg dose of ciprofloxacin in the clinic, 4.3 μg/mL, and which corresponds to 14× MIC in our study). After 72 h post infection, they observed substantial bacteria-killing and multiple log increases in PFUs. Only a few PAO1 mutants were isolated following treatments, with all of these mutants’ ciprofloxacin sensitive and all resistant to some or all phages. The time of treatment and these resistance results together are highly suggestive that at the start of treatments, which was six hours following epithelial cell infection with the bacteria, PAO1 cells were no longer in a planktonic state but instead had formed aggregates with biofilm-like properties. As with the biofilm studies discussed above, these bacteria therefore possibly exhibited increased tolerance to ciprofloxacin. In addition or alternatively, ciprofloxacin can positively diffuse and is taken up actively by Calu-3 epithelial cells [160,161], possibly lowering effective ciprofloxacin concentrations. MICs were determined using MHB.
