# Supplementary material for: Friends or Foes? Rapid Determination of Dissimilar Colistin and Ciprofloxacin Antagonism of Pseudomonas aeruginosa Phages
Source: Pharmaceuticals (Basel). 2021 Nov 15;14(11):1162. doi: 10.3390/ph14111162 (PMC8624478; doi:10.3390/ph14111162)
Supplement: Supplementary file 1 [file pharmaceuticals-14-01162-s001.zip › pharmaceuticals-1445156-supplementary.pdf]

SUPPLEMENTARY DATA

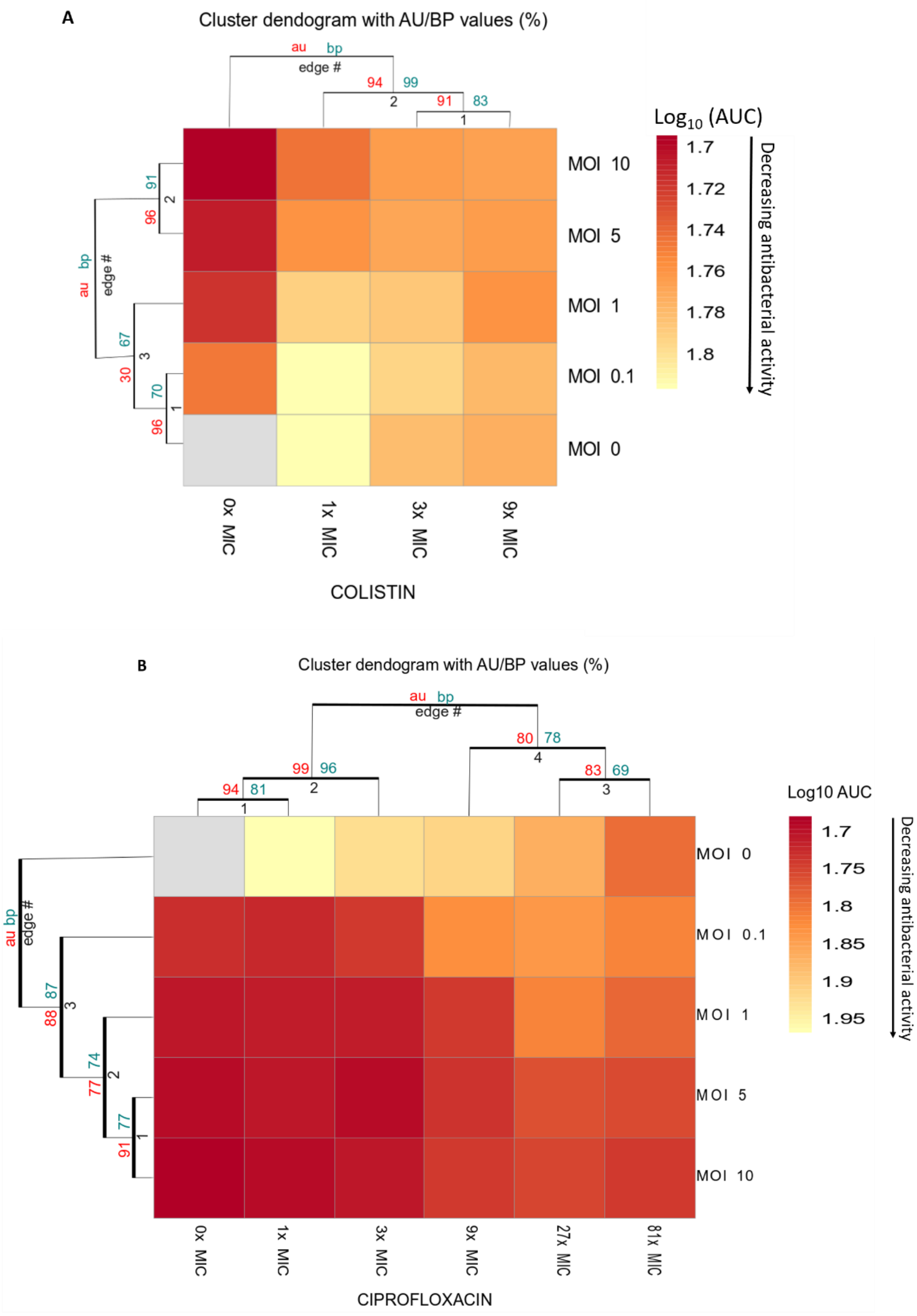

Figure S1. Heat map analyses of PEV2 infection with colistin (A) and ciprofloxacin (B). Heat maps were created based on $\log_{10}$(AUC). Calculations in each panel are based on 6 replicates from 2 independent experiments. [*] $p<0.005$, ANOVA; [AU] approximately unbiased *p*-value; [BU] bootstrap probability value, [edge #] *p*-values (%).

Spot test analysis employing different isogenic *P. aeruginosa* PAO1 surface mutants revealed that LPS, specifically O-specific antigen (OSA, formerly termed B-band) serves as an essential bacterial cell surface structure for successful phage PEV2 adsorption, and type IV pili in case of phage LUZ19 and ϕKMV. (Table S2).

Table S1. Phage receptor identification using isogenic *P. aeruginosa* PAO1 surface mutants.

	**Bacterial strain**	**Phenotype**	**Origin**	**Sensitivity to PEV2**	**Sensitivity to LUZ19**	**Sensitivity to ϕKMV**
**Wild Type**	PAO1 Krylov	Wild type	Queen Astrid Military Hospital, Belgium, Jean-Paul Pirnay	+	+	+
	PAO1 (ATCC 15692)	Wild type	American Type Culture Collection, Jean-Paul Pirnay	+	+	+
	PAO1	Isogenic wild type	Harvard University	+	+	+
**Type IV deficient**	PAO1 Pirnay	Wild type with inactive type IV pili	Queen Astrid Military Hospital, Belgium, Jean-Paul Pirnay	+	-	-
	PAO1*ΔpilA*	Lack of type IV pili	University of Washington, USA Matthew R. Parsek	+	-	-
**Flagella deficient**	PAO1*ΔfliC*	Lack of flagella	University of Calgary, Canada, Joseph Harrison	+	+	+
**Flagella & Type IV deficient**	PAO1*ΔfliCΔpilA*	Lack of flagella; lack of type IV pili	University of Calgary, Canada, Joseph Harrison	+	-	-
**LPS variants**	PAO1Δ*gmd* (CPA-)	Lack of *gmd* gene responsible for the biosynthesis of GDP-D-Rha, the nucleotide sugar precursor for CPA	University of Guelph, Canada, Joseph S Lam[58]	+	+	+
	PAO1Δ*rmlC* (OS-)	Lack of *rmlC* gene, responsible for TDP-L-Rha biosynthesis that lead to defective core OS truncated at the	University of Guelph, Canada, Joseph S Lam[58]	-	+	+

	Bacterial strain	Phenotype	Origin	Sensitivity to PEV2	Sensitivity to LUZ19	Sensitivity to ϕKMV
		Rha^A and Rha^B residues in the two glycoforms of the OS				
	PAO1Δ*wbpW* (CPA-)	Lack of *wbpW* gene that encode enzymes responsible for the biosynthesis of GDP-D-Rha, the nucleotide sugar precursor for CPA	The Ohio State University, USA, Daniel Wozniak	+	+	+
	PAO1Δ*wzy* (OSA-)	Lack of OSA polymerization of LPS. LPS consists OS and one OSA. CPA is intact	University of Guelph, Canada, Joseph S Lam[58]	-	+	+
	PAO1Δ*waaL* (CPA-, OSA-)	Lack of WaaL ligating O-polymer to core-lipid A; LPS is devoid of CPA and OSA, semi rough (SR-LPS, or core-plus-one O-antigen)	University of Guelph, Canada, Joseph S Lam[58]	-	+	+
	PAO1Δ*algC*	Lack of *algC* required for CPA, core oligosaccharide, and alginate biosynthesis	Emory University, USA, Joanna Goldberg	-	+	+

"+" indicates: allows clear spot formation (sensitive strain to phage treatment and presumably phage adsorption);
"-": indicates: does not allow spot formation (not sensitive strain to phage treatment and presumably phage adsorption);
CPA: LPS common polysaccharide antigen (formerly termed A-band);
GDP-D-Rha- nucleotide precursor for CPA;
L-Rha: L-rhamnose;
LPS: bacterial lipopolysaccharide;
OS: the LPS core oligosaccharide;
OSA: O-specific antigen (formerly termed B-band);
Rha^A and Rha^B : residues in the two glycoforms of the core oligosaccharide of LPS;
TDP-L-Rha: a sugar donor and core oligosaccharide of LPS

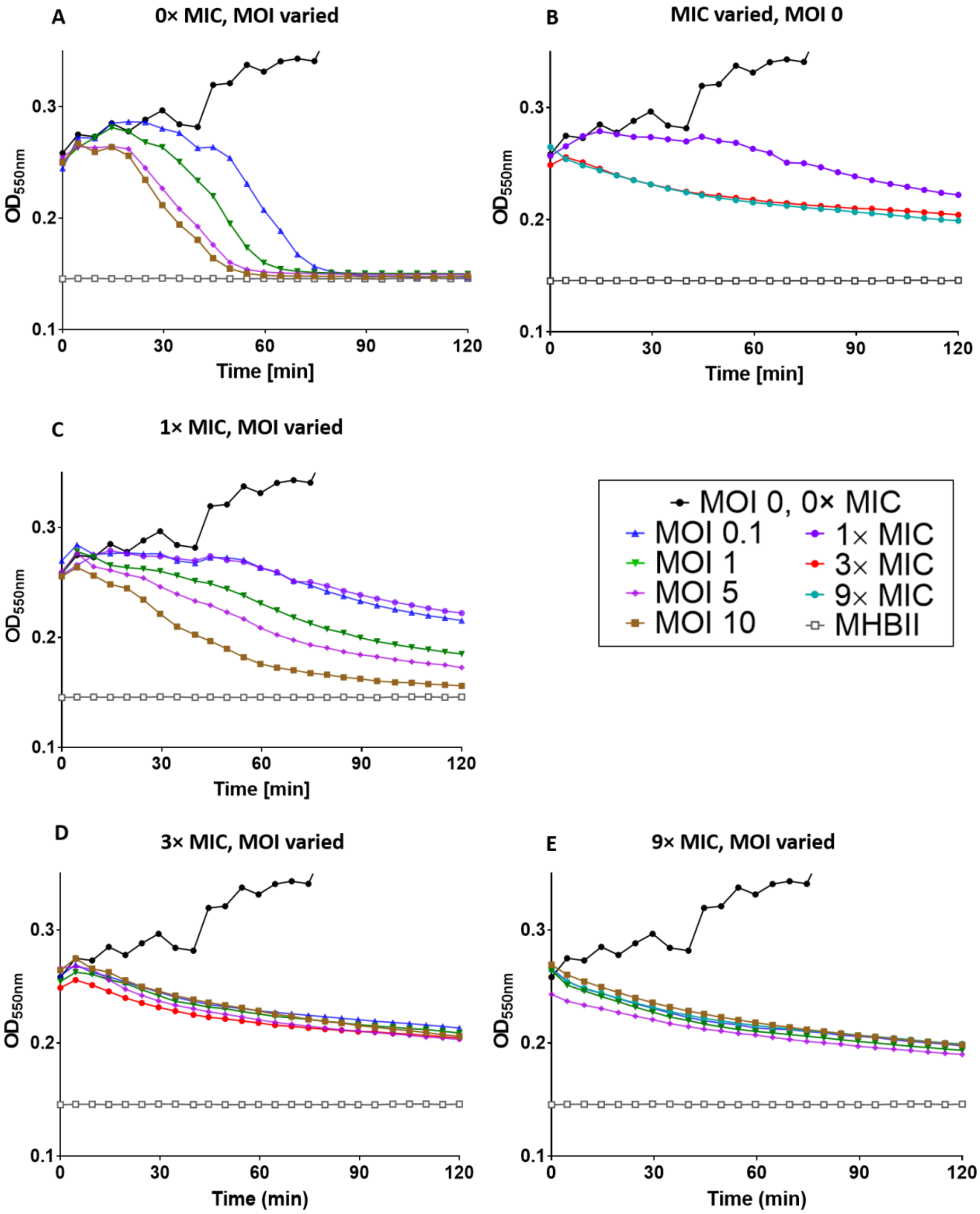

Figure S2. High antagonism of colistin on LUZ19 infection activities. (A) Phage LUZ19 behavior in the absence of colistin and impact on *P. aeruginosa* PAO1 cultures. **(B)** Impact of different concentrations of colistin on *P. aeruginosa* PAO1 culture without phage. **(C-E)** Impacts of 1×, 3×, and 9× MIC concentrations of colistin, respectively, at various MOIs of phage LUZ19. A key is provided in the black frame. See Appendix A for description of the explicit colistin concentrations used and Appendix B for interpretation of lysis profile results. A single representative experiment is shown.

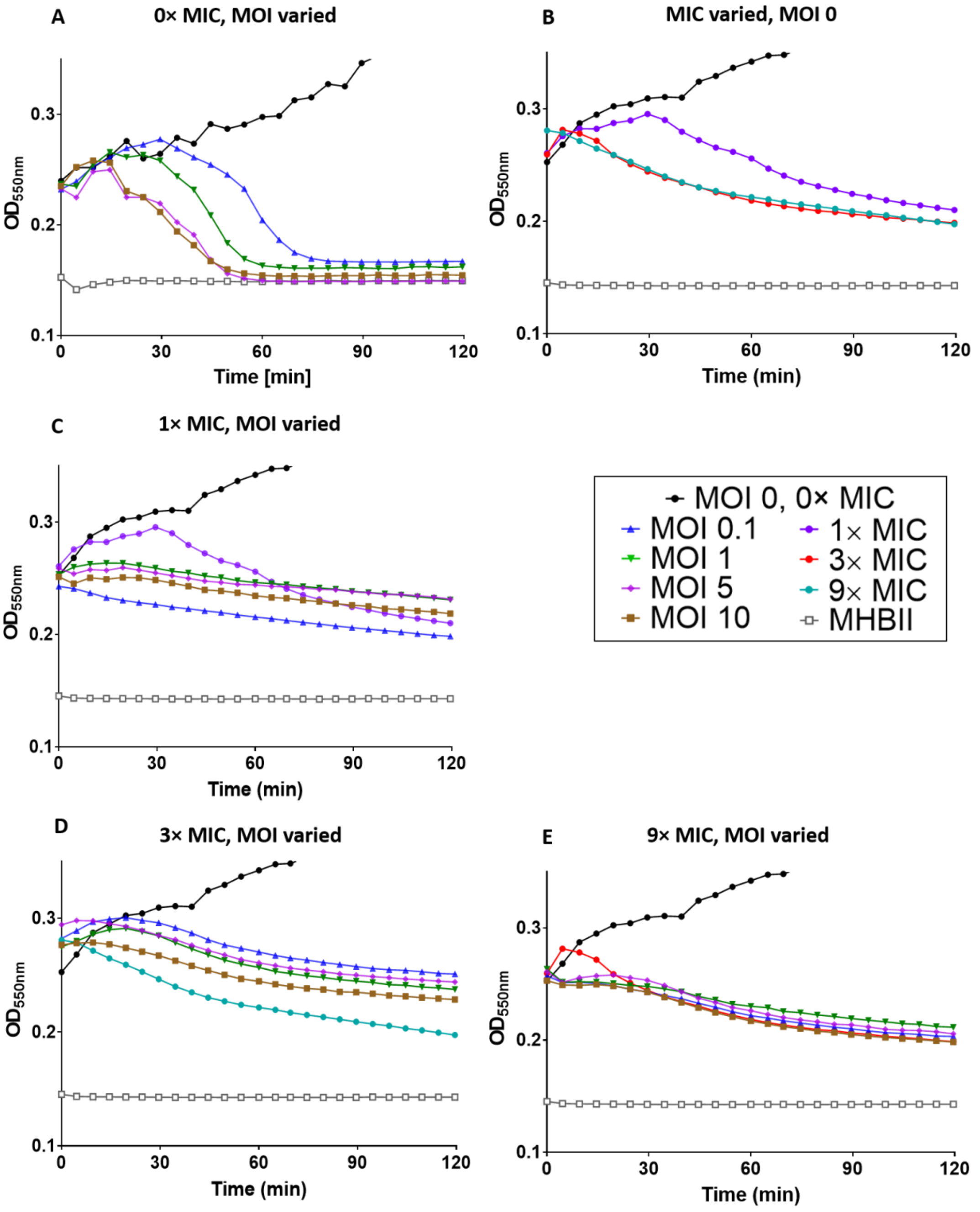

Figure S3. High antagonism of colistin on ϕKMV infection activities. See Figure S2 legend for details.

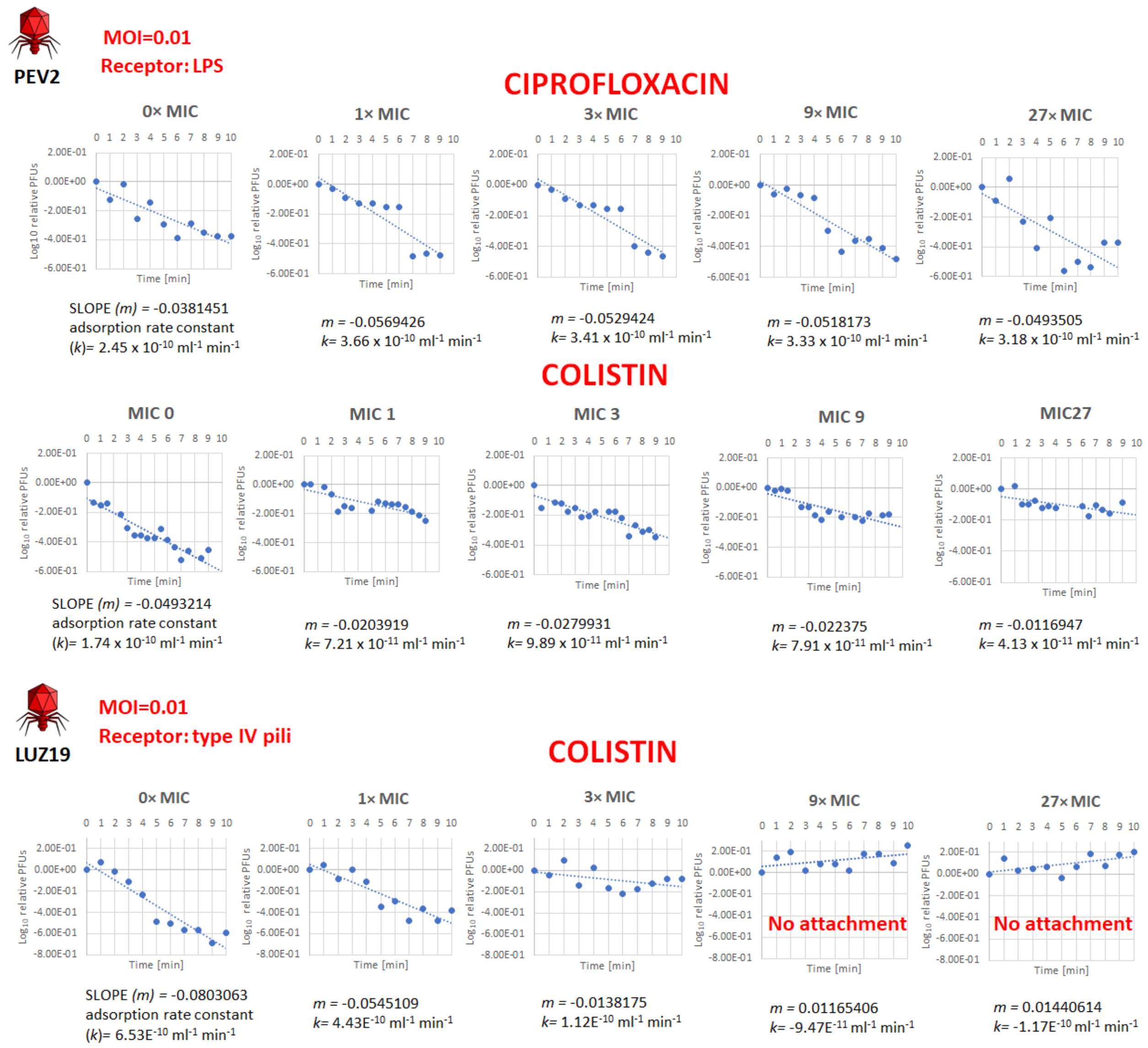

Figure S4. Phage adsorption curves reveals high antagonism of colistin. [*m*] slope, [*k*] adsorption rate constant, [MOI] multiplicity of infection, [LPS] lipopolysaccharide.

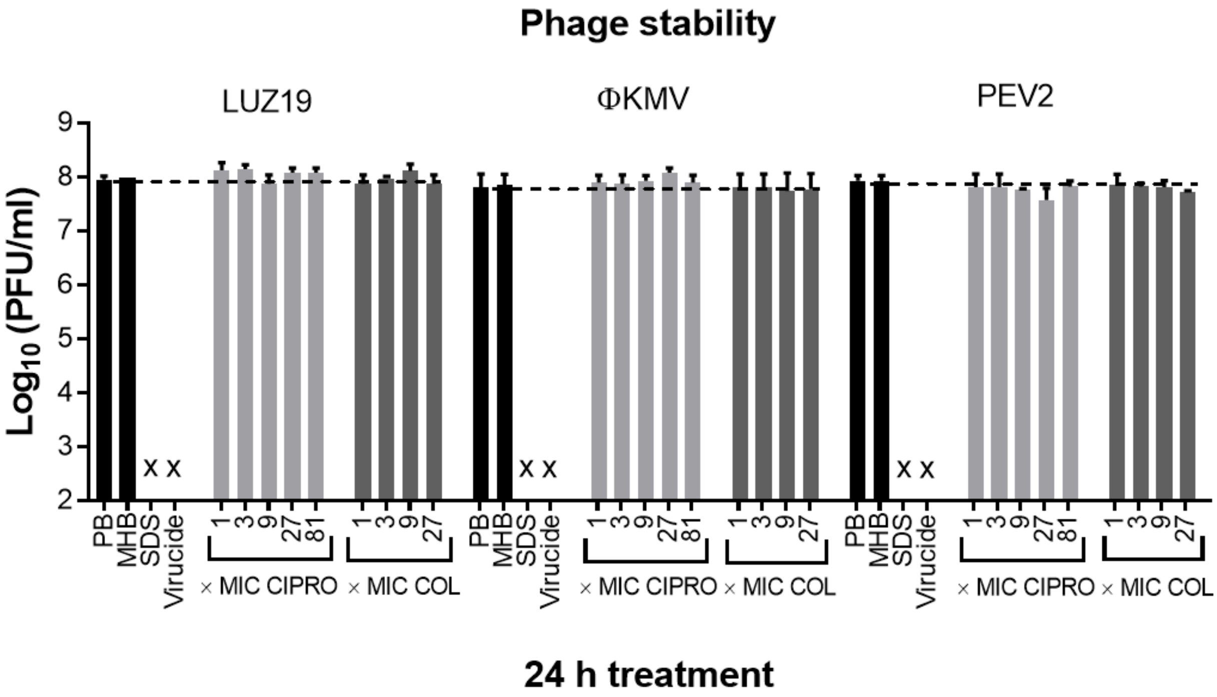

Figure S5. Phage stability in the presence of colistin and ciprofloxacin. [PFU] plaque-forming unit, [PB] phage buffer, [MHB] Mueller Hinton Broth cation adjusted, [SDS] 0.1% sodium dodecyl sulfate, [virucide] 7.5% w/v loose-leaf black Ceylon tea mixed with 4.3 mM $FeSO_4$, [X] results where detection of viable phage virions presence was not possible.

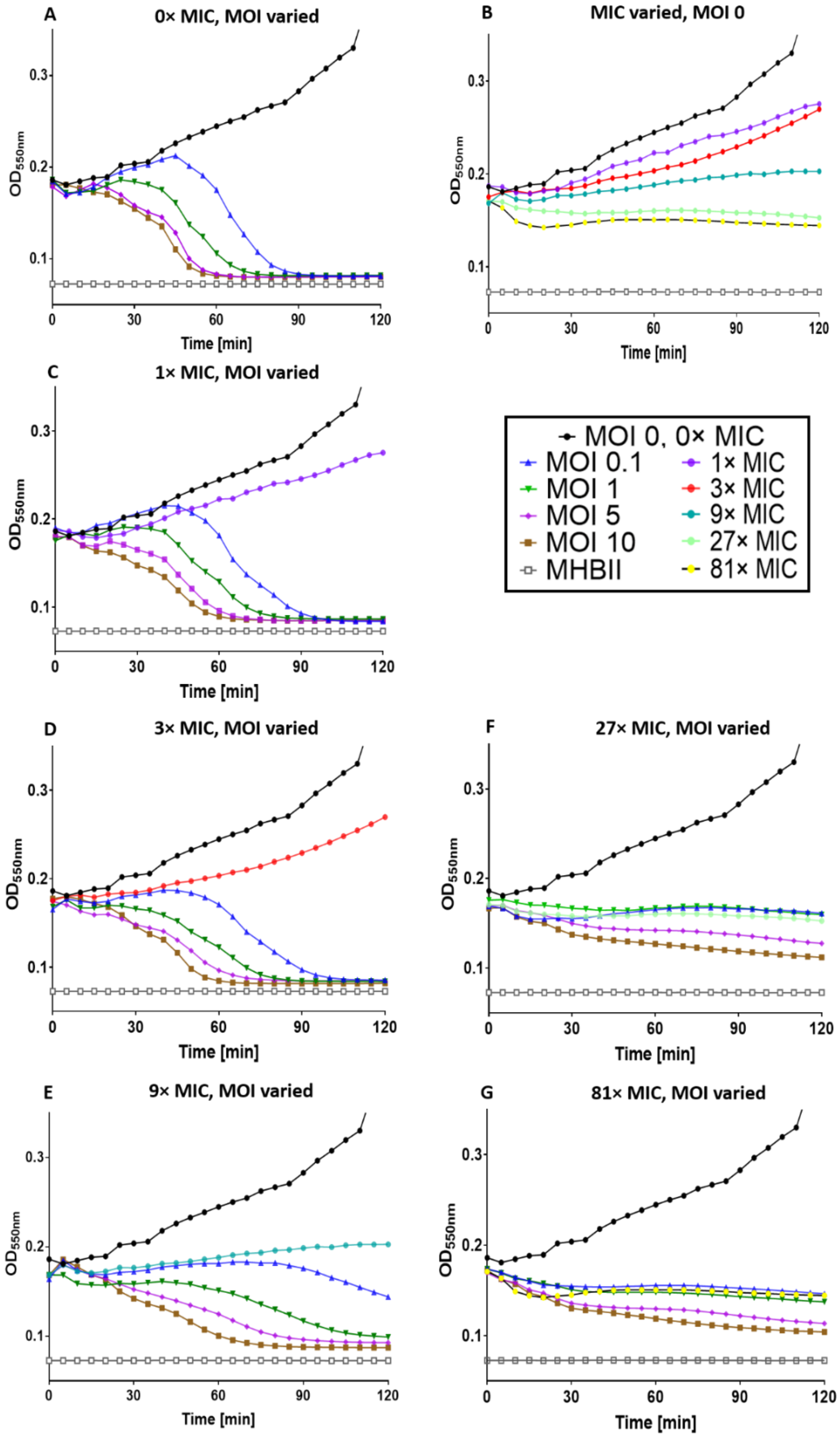

Figure S6. Low antagonism of ciprofloxacin on LUZ19 infection activities. See Figure S2 legend for details.

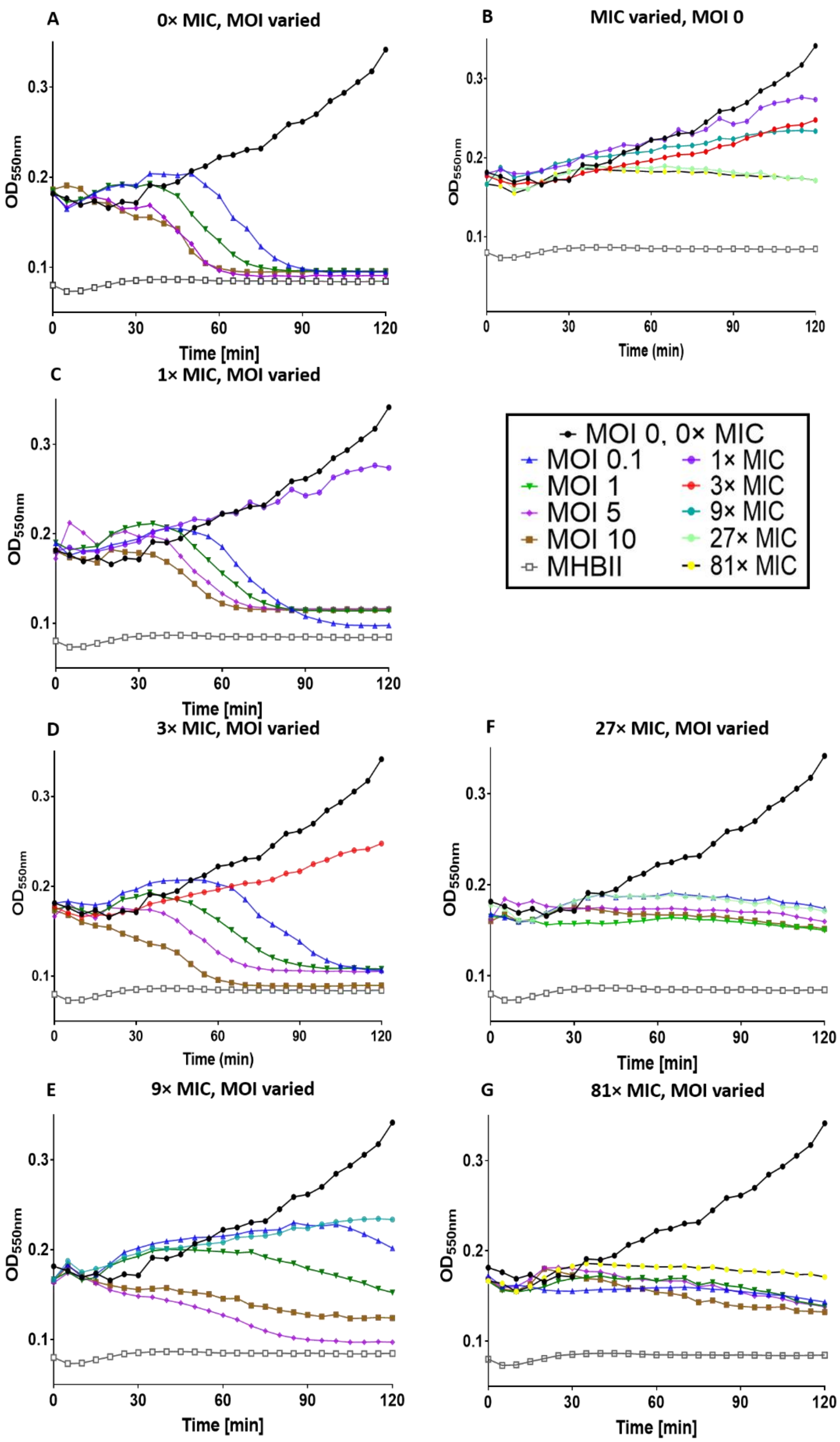

Figure S7. Low antagonism of ciprofloxacin on ϕKMV infection activities. See Figure S2 legend for details.
